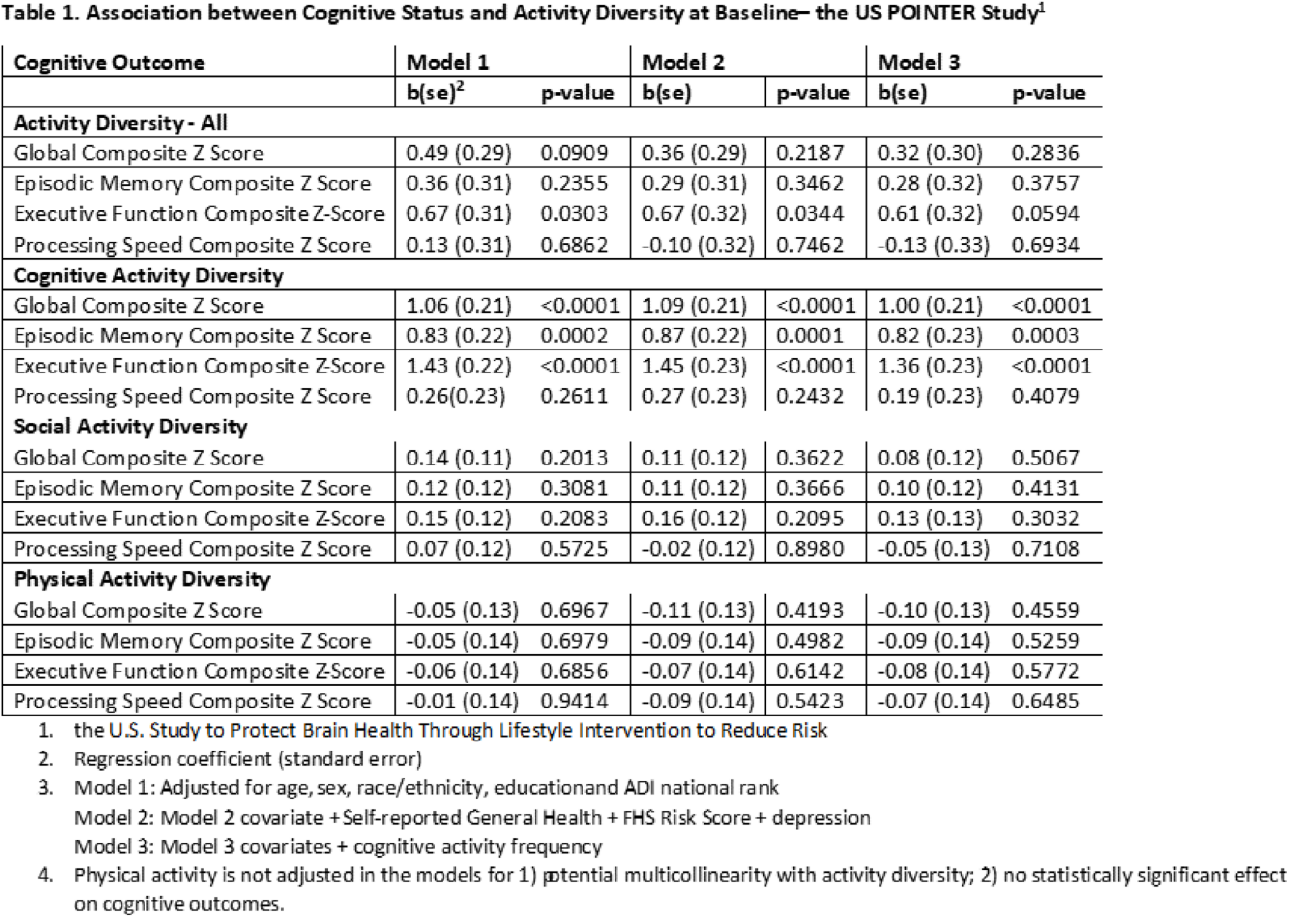# Activity Diversity and Its Association with Cognitive Function at Baseline in the U.S. POINTER Study

**DOI:** 10.1002/alz.086192

**Published:** 2025-01-09

**Authors:** Xiaoyan Leng, Marjorie Howard, Shannon Halloway, Sarah Tomaszewski Farias, Athene KW Lee, Kathryn V Papp, Laura D Baker, Heather M Snyder, Mark A. Espeland

**Affiliations:** ^1^ Wake Forest University School of Medicine, Winston‐Salem, NC USA; ^2^ Rush University College of Nursing, Chicago, IL USA; ^3^ University of California, Davis, Davis, CA USA; ^4^ Warren Alpert Medical School of Brown University, Providence, RI USA; ^5^ Butler Hospital, Providence, RI USA; ^6^ Massachusetts General Hospital, Department of Neurology, Harvard Medical School, Boston, MA USA; ^7^ Wake Forest University, Winston‐Salem, NC USA; ^8^ Alzheimer’s Association, Chicago, IL USA; ^9^ Wake Forest University School of Medicine, Winston Salem, NC USA

## Abstract

**Background:**

Recent cross‐sectional and longitudinal studies have shown that higher activity diversity ‐ defined as richness and evenness of daily activities are beneficial to older adults’ cognitive performance. This benefit is independent of frequency or level of cognitive challenge or physical activity.

**Method:**

A total of 1688 participants from the U.S. Study to Protect Brain Health through Lifestyle Intervention to Reduce Risk with physical, mental and social activities and cognitive outcomes (global, episodic memory, executive function and processing speed composite scores) measured at the baseline were included in this analysis. The sample was characterized by a mean age of 68.4 (SD: 5.2), 66.9% female, 29.6% less than college education and 33.6% non‐white. A total of 41 activities (11 mental, 21 physical and 10 social activities) are included to evaluate total, mental, social and physical activity diversity based on Shannon’s entropy (0 as no diversity and 1 as full diversity). Walking to do errands (e.g., take children to school or to/from a store) is considered both physical and social. A sequential multiple linear regression models were used to examine associations between activity diversities and cognitive outcomes, adjusted for (model 1) age, sex, race/ethnicity, education, area deprivation index national rank (ADI), and (model 2) additionally self‐reported general health, Framingham cardiovascular disease 10‐year Risk Score, depression (Geriatric Depression Scale total score) and finally (model 3) adding cognitive activity frequency (times/week).

**Result:**

Higher total activity diversity is associated with better executive function in models 1 and 2 (p <0.0344) and the association was attenuated when adding cognitive activity frequency as in model 3 (p = 0.0594). Total activity diversity is not associated with global cognition, or episodic memory or processing speed. Higher mental activity diversity is associated with higher global cognition (p<0.0001), episodic memory (p <0.0003) and executive function (p < 0.0001) in all three models, but not processing speed (p >0.2432).

**Conclusion:**

These results indicate that cognitive activity diversity is associated with cognitive function in older adults at risk of cognitive decline, which is independent of other risk factors and frequency of cognitive activities.